# GABA_B_ Receptor Signaling in the Dorsal Motor Nucleus of the Vagus Stimulates Gastric Motility via a Cholinergic Pathway

**DOI:** 10.3389/fnins.2019.00967

**Published:** 2019-09-12

**Authors:** Maureen T. Cruz, Ghazaul Dezfuli, Erin C. Murphy, Stefano Vicini, Niaz Sahibzada, Richard A. Gillis

**Affiliations:** Department of Pharmacology and Physiology, Georgetown University Medical Center, Washington, DC, United States

**Keywords:** vagus, motility activation, gastric, acetylcholine, vagotomy, NANC (non-adrenergic, non-cholinergic)

## Abstract

Central nervous system regulation of the gastric tone and motility is primarily mediated via preganglionic neurons of the dorsal motor nucleus of the vagus (DMV). This is thought to occur by simultaneous engagement of both independent excitatory and inhibitory pathways from the DMV and has been proposed to underlie the opposing effects seen on gastric tone and motility in a number of *in vivo* models. Contrary to this view, we have been unable to find any evidence for this “dual effector” pathway. Since this possibility is so fundamental to how the brain-gut axis may interact in light of both peripheral and central demands, we decided to explore it further in two separate animal models previously used in conjunction with GABA_B_ signaling to report the existence of a “dual effector” pathway. Using anesthetized rats or ferrets, we microinjected baclofen (7.5 pmol; *n* = 6), a GABA_B_ agonist into the DMV of rats or intravenously administered it (0.5 mg/kg; *n* = 4) in ferrets. In rats, unilateral microinjection of baclofen into the DMV caused a robust dose-dependent increase in gastric tone and motility that was abolished by ipsilateral vagotomy and counteracted by pretreatment with atropine (0.1 mg/kg; IV). Similarly, as microinjection in the rats, IV administration of baclofen (0.5 mg/kg) in the ferrets induced its characteristic excitatory effects on gastric tone and motility, which were blocked by either pre- or post-treatment with atropine (0.1 mg/kg; IV). Altogether, our data provide evidence that the gastric musculature (other than the gastric sphincters) is regulated by a “single effector” DMV pathway using acetylcholine.

## Introduction

Vagal regulation of the gastric musculature is thought to occur by simultaneous engagement of independent excitatory and inhibitory pathways that originate from the brainstem’s dorsal motor nucleus of the vagus (DMV; Wood, 2012). This source of vagal outflow to the stomach is controlled largely by sensory information from the gastrointestinal (GI) tract and represents a key component of the vago-vagal reflex (Rogers and Hermann, 2012). The vagal inhibitory pathway, a non-adrenergic, non-cholinergic pathway (NANC), is thought to employ nitric oxide (NO), vasoactive intestinal peptide (VIP) and adenosine as its neurotransmitters; whereas, the excitatory pathway exerts its effect by cholinergic transmission (Rogers and Hermann, 2012). While both pathways originate from the DMV, they can be simultaneously engaged by neurons originating from the nucleus tractus solitarius (NTS). This concept of “dual vagal effectors” controlling cholinergic and NANC input to the stomach has been proposed to form the basis of the opposing effects seen on the gastric musculature (Rogers and Hermann, 2012).

Evidence for the concept of two opposing efferent DMV pathways controlling gastric motility stems from studies on the esophageal-gastric relaxation reflex (Rogers et al., 1999, 2003), cholecystokinin (CCK; Lloyd et al., 1993), gastric accommodation reflex (Takahashi and Owyang, 1997), oxytocin (Holmes et al., 2013), corticotrophin-releasing factor (Lewis et al., 2002) (CRF), and baclofen (Andrews et al., 1987; Browning and Travagli, 2001). Contrary to the above studies, we have been unable to obtain any evidence for the existence of ‘functional’ parallel excitatory and inhibitory DMV vagal pathways that influence gastric smooth muscle in the rat. To this end, we have used four different stimuli to functionally engage the DMV and to assess their effect on gastric motility. These include esophageal distention (Ferreira et al., 2002, 2005), intravenous administration of CCK (Shi et al., 2005), glucose (Shi et al., 2005), and nicotine (Ferreira et al., 2002). Although each of these stimuli induced vagally mediated gastric inhibition of motility (Ferreira et al., 2002, 2005; Shi et al., 2005); this inhibition stemmed from suppression of the excitatory cholinergic DMV pathway, and not by activation of postganglionic NANC transmission (Ferreira et al., 2002, 2005; Shi et al., 2005). Similarly, we were unable to obtain evidence that oxytocin and CRF inhibit gastric smooth muscle by activation of a NANC pathway originating from the DMV (Cruz et al., 2007).

Nevertheless, there is additional evidence that NANC transmission may be responsible for the inhibition of gastric motility elicited from the DMV. This evidence is based on studies of the GABA_B_ agonist, baclofen. In rat brainstem slices, baclofen has been reported to act directly on neurons in the nucleus tractus solitarius (NTS) to produce a membrane hyperpolarization (Brooks et al., 1992); thus, most likely exciting DMV neurons by disinhibition. At the same time, baclofen also exerts a direct inhibitory effect on DMV neurons (Browning and Travagli, 2001). The baclofen-induced inhibition is thought to selectively affect inhibitory NANC neurons, thereby increasing gastric corpus and lower esophageal sphincter pressures (Browning and Travagli, 2001). In an *in vivo* ferret preparation, intravenous administration of baclofen increases gastric corpus pressure in the presence of cholinergic and sympathetic inhibition (Andrews et al., 1987). This increase in gastric pressure is thought to be due to a baclofen-induced decrease in the tonic vagal drive to NANC inhibitory neurons (Andrews et al., 1987). The purpose of our study was to explore the possibility of whether gastric motility effects induced by baclofen were indeed due to inhibitory NANC transmission. Using *in vivo* rat and ferret models, we report here that microinjection of baclofen into the DMV of the rat, and IV administration in the ferret increases gastric motility only by activation of an excitatory vagal pathway.

## Materials and Methods

### Animals and Surgical Preparation

Experiments were performed on male Sprague-Dawley rats (250–350 g; Taconic, MD) and on male sable ferrets (700–900 g; Harlan, IN) in accordance with the National Institute of Health guidelines for the use of animals in research and with the approval of the Animal Care and Use Committee of Georgetown University. Animals were fasted overnight (∼12 h), while water was provided *ad libitum*. All experiments were performed in anesthetized animals; the choice of anesthetic was predicated on that used in earlier studies to determine the presence of a NANC inhibitory drive to the stomach (Andrews et al., 1987; Krowicki et al., 1999; Krowicki and Hornby, 2000).

In all experiments, rats were anesthetized with a mixture of urethane and α-chloralose (800 mg/kg + 60 mg/kg; IP) dissolved in 3 ml of 0.9% saline. In ferrets, anesthesia was constituted with urethane alone (1.5 g/kg; IP) that was dissolved in 2 ml of 0.9% saline. Body temperature was maintained at 37°C + 1°C with an infrared heating lamp. After a surgical depth of anesthesia was confirmed, a tracheotomy was performed to allow for an endotracheal tube to be inserted so as to maintain a patent airway, and to provide artificial respiration if necessary. Next, the left carotid artery and the external right jugular vein were cannulated with polyethylene tubing (PE50) for monitoring blood pressure (BP) and for the systemic administration of drugs, respectively. To facilitate access to the vagus nerves, both cervical vagi were carefully isolated from the surrounding tissue, and silk suture loops were placed around each vagal trunk. This enabled us to section either one or both nerves, as dictated by the experimental protocol. BP was monitored via a pressure transducer (using a DC-coupled 100 Hz low-pass filter; sensitivity: 5 μV/V/mmHg), which was coupled to a bridge amplifier and a data acquisition system (PowerLab, ADI Instrument, Colorado Springs, CO, United States).

To monitor gastric tone and phasic motility, a low-compliance latex balloon (connected to PE-160 tubing) was inserted into the stomach via the fundus and positioned toward the corpus/antrum area. The balloon was inflated with warm distilled water (3–5 ml for rats and 8–10 ml for ferrets) to produce a global distention of the stomach with a baseline intragastric pressure (IGP) of 8–12 mmHg. Preliminary experiments indicated that baseline IGP between 8–12 mmHg resulted in a maximal contractile response when the DMV was excited by 30 nl of 16.7 mM L-glutamate (i.e., 500 pmol). The tubing attached to the balloon was connected to a pressure transducer (DC-coupled 20 Hz low-pass filter; sensitivity: 5 μV/V/mmHg), which was connected via a bridge amplifier to the same data acquisition system as used by the BP transducer.

### Microinjection Procedure and Verification of Microinjection Sites

Each anesthetized rat was placed in a prone position in an animal stereotaxic frame (Kopf Instruments, Tujunga, CA, United States). Before stereotaxic surgery, each animal was pretreated with dexamethasone (0.8 mg, SC) to minimize swelling of the brain. A skull-base partial craniotomy was performed to expose the dorsal medulla. To gain access to the entire rostrocaudal extent of the DMV that underlies the NTS, the cerebellum was retracted, and the underlying dura was cut and reflected to expose the NTS underneath.

A double-barrel glass micropipette (tip Ø = 30–70 μm; Fredrick Haer, New Brunswick, ME, United States) was inserted into the DMV using the calamus scriptorius (CS) as a reference point for calculating the coordinates for micropipette placement in the rat. Stereotaxic coordinates for microinjection into the DMV for the rat in reference to CS were: AP = 0.1–0.6 mm, ML = 0.3–0.6 mm, and DV = 0.5–0.9 mm.

The precise location of the DMV was assessed by microinjecting L-glutamate (500 pmol/30 nl) into the stereotaxically designated site and noting an increase in IGP. This dose of L-glutamate is based on a dose-response curve derived from microinjection studies in the rat using gastric motility as the end point of an effect (Ferreira et al., 2000).

All microinjections in the brainstem were made with double-barreled glass micropipette (tip Ø 30–70 μm; Fredrick Haer, New Brunswick, ME, United States) that was angled at 30° from the perpendicular. Drugs were loaded and ejected from each barrel by negative or positive pressure, respectively, through a 5 ml syringe that was connected via a PE-50 polyethylene tubing. Drug injections into the DMV were administered manually within 5 to 10 s in a 30 nl volume, as monitored by a calibration tape affixed to the pipette. For intravenous administration, rats received drugs while in the stereotaxic frame, whereas ferrets were in a supine position and not placed into the stereotaxic frame. Drugs were delivered via a perfusion pump over 1 min.

At the end of each experiment, the animal was euthanized with an overdose of pentobarbital. The brain was removed and fixed in a solution of 4% paraformaldehyde and 20% sucrose for at least 24 h. It was then cut on a cryostat into 50 μm-thick coronal serial sections and stained with 0.5% neutral red. Using bright-field microscopy, the locations of the microinjection sites were determined, photographed and camera lucida drawings were made of the brainstem sections that contained them. The location of microinjection sites for the rat was noted in relation to nuclear groups using the atlas of Paxinos and Watson (1998).

### Experimental Protocol

In all experiments, a stable baseline IGP and BP recordings were obtained for at least 10 min before any experimental intervention was initiated. The micropipette was inserted unilaterally, and the animal was allowed to stabilize for at least 2 min thereafter. In all microinjection studies, L-glutamate (30 nl of 16.7 mM solution; 500 pmol total) was used as a pharmacological tool to identify the DMV location. A minimum of 10 min was allowed to elapse between L-glutamate injections. [**Note:** this time interval has been empirically determined by us to be sufficient to obtain reproducible gastric motility responses.] Following a repeat microinjection of L-glutamate and a resultant stable response similar to the first one, baclofen was microinjected into the DMV. After establishing the effect of baclofen microinjected into the DMV, vagotomy and/or systemic administration of atropine methyl bromide was performed before repeating a second microinjection of baclofen.

The protocol used for IV studies of baclofen in the ferret is described in the “Results” section. The starting dose of baclofen (75 pmol) was chosen based on our ventrolateral NTS studies in the rat (Wasserman et al., 2002). The dose of baclofen (0.5 mg/kg) chosen for the ferret studies is based on its previous use in the ferret (Andrews et al., 1987). The dose of atropine methyl bromide (0.1 mg/kg) chosen for both the rat and ferret was based on studies that show a complete block of muscarinic receptors in the periphery (Flacke and Gillis, 1968). Moreover, atropine methyl bromide, a quaternary ammonium derivative of atropine was also chosen as it does not readily cross the blood-brain barrier due to its a charged nature (Khavari and Maickel, 1967).

### Data Analysis

Changes in gastric tone and phasic motility in response to microinjection of a drug were compared to a 3 min baseline recording and analyzed using Lab Chart (ADI Instruments, Colorado Springs, CO, United States). Each baseline recording was divided into a 1 min segment, averaged and compared to the drug effect. The baseline gastric tone was defined as the global tone of the stomach, whereas the phasic motility (measured by AUC) was an indicator of the phasic motility of the gastric smooth muscle. Changes in mean BP were also analyzed. The mean BP over a 3 min baseline period was compared to the mean BP following microinjection of a drug.

In experiments where a drug was injected twice (under identical experimental conditions), the two responses were averaged to determine the mean response. Data are reported as mean ± SEM. A one-sample *t*-test was performed to test if a group’s mean was significant from zero. A paired *t*-test was performed when animals served as their own controls. An unpaired *t*-test (independent samples) was performed on data from a separate control and experimental groups. A comparison among three or more different groups was made by a one-way repeated measures ANOVA test followed by a *post hoc* Newman-Keuls test for specific group comparisons. In all cases, *p* < 0.05 was the criterion used to denote statistical significance.

### Drugs

Drugs used for the studies were: urethane, α-chloralose, dexamethasone sodium phosphate, L-glutamate, baclofen HCL, and atropine methyl bromide. All drugs were purchased from Sigma-Aldrich (St. Louis, MO, United States) with the exception of dexamethasone, which was purchased from American Regent Laboratories (Shirley, NY, United States). All drug solutions were constituted in 0.9% saline (pH 7.2–7.4).

## Results

Data obtained with the GABA_B_ receptor agonist baclofen provides some of the positive evidence in support of a DMV cholinergic/NANC vagal inhibitory pathway to the stomach (Andrews et al., 1987; Browning and Travagli, 2001).

### Microinjection of Baclofen in the DMV (Rat)

**FIGURE 1:**
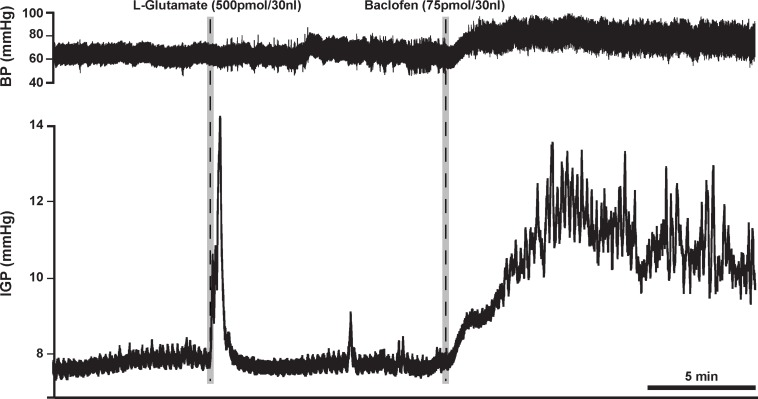
Microinjection of baclofen (75 pmol) into the DMV of the rat causes a robust increase in gastric tone and motility, which can persist for more than 2 h. L-glutamate, whose effect lasts for approximately 5 min, is initially microinjected to establish that the subsequent microinjection will be in the DMV and not in the overlying NTS; a site known to cause inhibition of gastric tone and motility (Herman et al., 2009).

Based on the aforementioned findings (Andrews et al., 1987; Browning and Travagli, 2001), we expected that microinjection of baclofen into the DMV of rats would produce an increase in gastric tone and motility either by attenuating tonic vagal drive to NANC inhibitory neurons to the stomach (Andrews et al., 1987) or by directly inhibiting NANC neurons in the DMV (Browning and Travagli, 2001). Indeed, microinjection of our starting dose of 75 pmol baclofen into the intermediate region of the DMV did evoke a significant robust increase in IGP. On average, this increase in IGP was 2.9 ± 0.5 mmHg from baseline control (Figure 1 and Table 1). As illustrated in Figure 1, microinjection of baclofen into the DMV had a long-lasting effect (>2 h) on both tone and motility. In contrast, microinjection of the vehicle for baclofen into the DMV had no significant effect on IGP or motility (**data not shown**).

**TABLE 1:** Changes in IGP, gastric motility (AUC) and mean arterial pressure (BP) produced by unilateral microinjection of baclofen into the DMV of the rat.

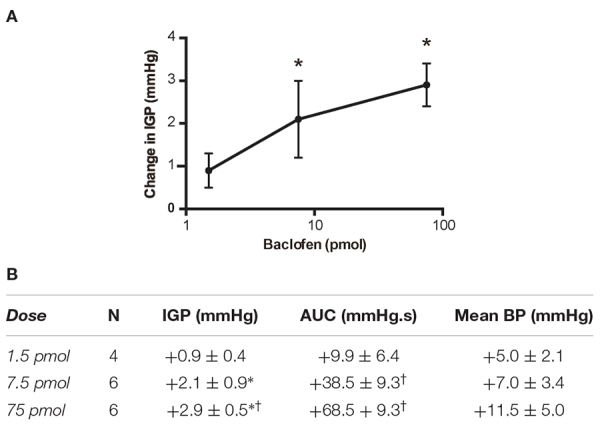

***(A)** Dose-response curve showing changes in IGP. **(B)** Summary of the baclofen-induced changes in gastric tone and motility, and in blood pressure elicited from the DMV. Values are means + SEM; ^∗^*p* < 0.05 ANOVA; ^†^*p* < 0.05, one sample *t*-test.*

**FIGURE 2:**
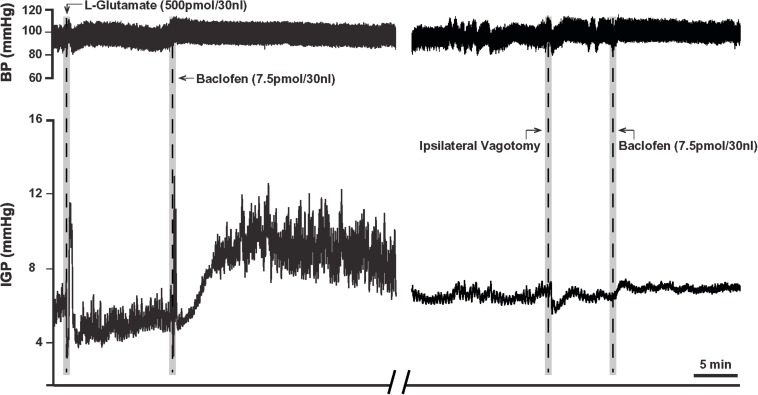
Microinjection of baclofen (7.5 pmol) into the DMV of the rat. **(Left)** Representative recording showing baclofen causes a slow rise in gastric tone followed by an increase in motility, which is reproducible after one-hour intervals. **(Right)** Following ipsilateral vagotomy, baclofen fails to induce its characteristic effects on gastric tone and motility in the same animal. Since the DMV projects ipsilaterally to the stomach, failure to elicit a response following ipsilateral vagotomy confirms the location of the microinjection site to be in the nucleus.

Due to the long time-course of action of the 75 pmol baclofen dose, it was deemed unsuitable as reliable responses with a second injection of the drug could not be replicated. This was particularly troublesome after experimental interventions such as ipsilateral vagotomy and atropine methyl bromide treatment. Hence, to study the effects of baclofen in the DMV before and after these experimental interventions, we reduced the dose to 7.5 pmol. At this dose, baclofen also produced a significant increase in IGP and gastric motility, which could be reliably repeated after 1 h (Figure 2 and Table 1). The time to reach a plateau phase of the increase in IGP was 4.2 ± 1.6 min after administration.

A third dose of baclofen (1.5 pmol) was also tested in four rats. The changes in IGP and gastric motility were not statistically significant (Table 1). Because of the modest time-course of action of the 7.5 pmol baclofen dose on IGP, and its repeatability, we selected it to test for activation of neurons in the DMV that comprise the vagal inhibitory NANC pathway.

Prior to determining the presence of the proposed parallel “dual effector” pathways (NANC and cholinergic) in the DMN (Andrews et al., 1987; Yamasaki et al., 1991; Lloyd et al., 1993; Rogers et al., 1999, 2003; Browning and Travagli, 2001; Lewis et al., 2002; Holmes et al., 2013) using baclofen, we first chose to determine if the drug’s effect on IGP was mediated by the vagus nerve. To this end, 7.5 pmol baclofen was microinjected into the DMV before and after ipsilateral vagotomy. [**Note:** ipsilateral vagotomy is done to confirm that a drug’s effect is induced from the DMV and not from the overlying NTS, which requires a bilateral vagotomy]. As can be seen in Figure 2 and Table 2, ipsilateral vagotomy prevented any effect of baclofen on gastric motility. All microinjections into the DMV were histologically verified, and are illustrated in Figure 4 along with a representative microinjection track.

**TABLE 2:** Changes in IGP, gastric motility (AUC), and mean arterial pressure (BP) produced by administration of baclofen before (pre-treatment), and after either ipsilateral vagotomy or atropine methyl bromide (post-treatment).

	**IGP (mmHg)**		**AUC (mmHg.s)**		**Mean BP (mmHg)**	
			
	**Response to baclofen**		**Response to baclofen**		**Response to baclofen**	
			
**Treatment N**	**Pre-treatment**	**Post-treatment**	**Pre-treatment**	**Post-treatment**	**Pre-treatment**	**Post-treatment**
*Ipsilateral Vagotomy* 4	+1.9 ± 0.74	+0.2 ± 0.2^∗^	+44.5 ± 27.1	+4.4 ± 2.5	+7.3 ± 4.0	+7.3 ± 0.3^†^
*Atropine 4*	+2.9 ± 0.80^†^	+0.2 ± 0.3^∗^	+44.3± 14.2	+11.7 ± 10.5	+8.6 ± 4.9	+7.3 ± 0.3^†^

*Values are means + SEM; ^∗^*p* < 0.05, paired *t*-test; ^†^*p* < 0.05, one sample *t*-test.*

**FIGURE 3:**
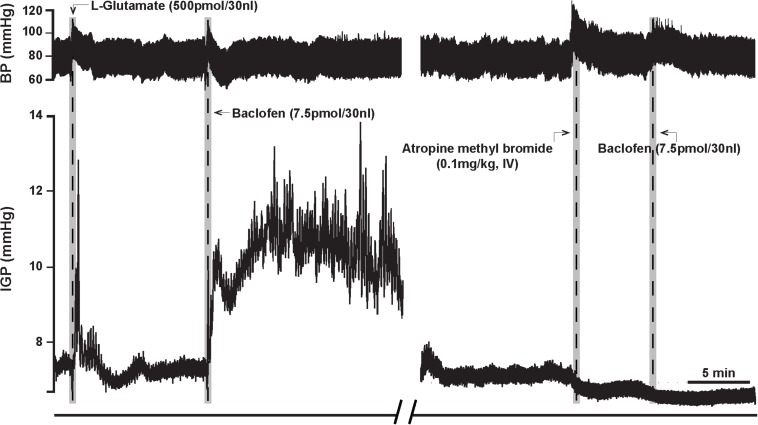
Atropine methyl bromide counteracts the effects of microinjection of baclofen (7.5 pmol) into the DMV of the rat. **(Left)** Representative recording showing the characteristic excitatory effects of baclofen on gastric tone and motility that is abolished by intravenous pre-treatment with atropine methyl bromide **(Right)**.

To determine whether baclofen microinjection in the DMV exerts its effect on gastric motility via an inhibitory or excitatory pathway (i.e., NANC or cholinergic, respectively), we debated if we should pretreat with a blocker of the neurotransmitter of enteric NANC neurons, or with an antagonist of the postganglionic cholinergic neurons. Since NANC neurons are thought to release several substances (e.g., NO, VIP, and adenosine; Rogers and Hermann, 2012), we chose to test the antagonist of the cholinergic neurons, namely atropine methyl bromide. As with ipsilateral vagotomy, pretreatment with atropine methyl bromide (0.1 mg/kg) totally prevented the gastric effects of baclofen (7.5 pmol) that was microinjected into the DMV (Figure 3 and Table 2). Since no gastric motility response to baclofen remained after atropine methyl bromide, we did not test antagonists of NANC neurotransmitters. Our experimental strategy to use atropine methyl bromide to demonstrate that baclofen increases gastric motility and tone through the activation of a vagally mediated cholinergic efferent pathway is analogous to the strategy used by Swartz et al. (2014) to study Ghrelin. Ghrelin microinjected into the dorsal vagal complex increased gastric motility and tone. Pretreatment with atropine methyl nitrate completely blocked the Ghrelin effect. Based on their data, Swartz et al. (2014) did not find it necessary to seek evidence for a role of vagally – mediated NANC pathway in the Ghrelin response.

**FIGURE 4:**
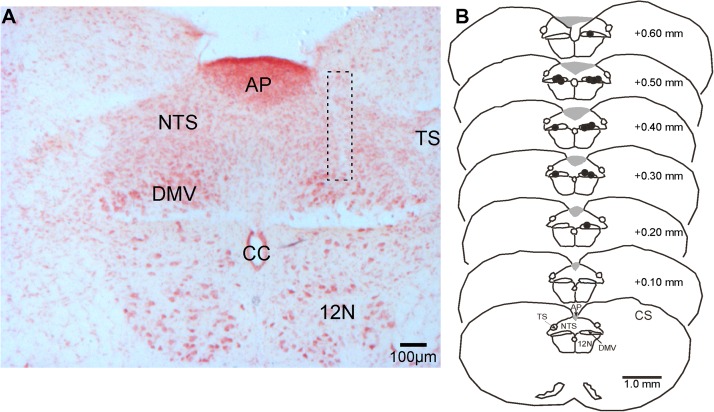
Representative photomicrograph of a pipette-track in the DMV and *camera lucida* drawings of brainstem sections showing the location of microinjection sites (black dots) in the nucleus in reference to the *calamus scriptorius* (CS). **(A)** Photomicrograph of a microinjection pipette-track targeting the DMV (dashed rectangle). **(B)** *Camera lucida* drawings of brainstem sections showing the distribution of microinjection sites (black dots) along the rostrocaudal extent of the DMV. 12 N, hypoglossal nucleus; AP, area postrema, CC, central canal; DMV, dorsal motor nucleus of the vagus; NTS, nucleus tractus solitarius; TS, solitary tract.

### Intravenous Administration of Baclofen (Ferret)

**FIGURE 5:**
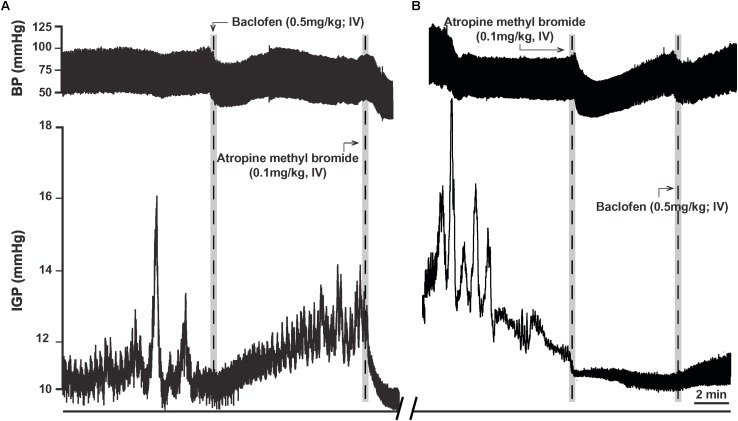
Effects of baclofen administration (0.5 mg/kg; IV) in the ferret on gastric tone and motility. **(A)** Representative recording of the excitatory effects of baclofen on gastric tone and motility, which is abolished by treatment with atropine methyl bromide (0.1 mg/kg; IV). **(B)** Representative recording showing baclofen fails to induce its characteristic effects on gastric tone and motility following pre-treatment with atropine methyl bromide.

In eight ferrets, we administered a dose of 0.5 mg/kg of baclofen (IV), as at this dose its effect on gastric motility has been reported to be in part due to inhibition of the cholinergic-NANC pathway (Andrews et al., 1987). In five ferrets, baclofen (0.5 mg/kg; IV) produced a slowly developing increase in gastric tone that averaged 1.6 ± 0.6 mmHg (*p* < 0.05; Table 2). The time to the peak of the response was 4.0 ± 0.9 min after IV injection of the drug (Figure 5A). Once the baclofen-induced increase in tone had plateaued and stabilized, atropine methyl bromide was infused (0.1 mg/kg; IV; *n* = 4). Atropine methyl bromide produced an immediate drop in gastric tone and returned the IGP to its baseline pressure (Figure 5A). The drop in IGP upon administration of atropine methyl bromide averaged −2.6 ± 0.6 mmHg (*p* < 0.05). Thus, atropine methyl bromide administration completely counteracted the rise in IGP produced by IV infusion of baclofen.

In another set of experiments, ferrets (*n* = 3) were pretreated with atropine methyl bromide (IV) prior to baclofen administration. Atropine methyl bromide produced an immediate inhibition of ongoing phasic contractility and decreased the IGP by −1.8 ± 0.1 mmHg. Subsequent administration of baclofen (0.5 mg/kg; IV) had no effect on gastric tone (Figure 5B). The changes in IGP and AUC were 0.1 ± 0.1 mmHg and 8.3 ± 5.6 mmHg.s, respectively. Thus, removal of the excitatory cholinergic drive to the stomach suppressed any increase in gastric tone produced by the administration of baclofen.

## Discussion

Our study focused on using the GABA_B_ receptor agonist baclofen to determine if the effects seen on gastric motility were due to the engagement of a DMV-NANC pathway that innervates the stomach. Since some of the key evidence for the existence of this pathway is derived from studies in the ferret (Andrews et al., 1987) and in the rat (Krowicki et al., 1999; Rogers et al., 1999, 2003; Krowicki and Hornby, 2000), our experiments were conducted in both species. Microinjection of baclofen in the DMV of the rat or its intravenous administration in the ferret failed to show any evidence for the role of a functional NANC pathway controlling gastric motility.

In the rat transverse brainstem slices, baclofen induces an outward whole-cell current and hyperpolarization in NTS neurons (Brooks et al., 1992). Similarly, bath application of baclofen induces an outward whole-cell current in 54% of DMV neurons that is in part was due to an increase in potassium conductance (Browning and Travagli, 2001). The synaptic blocker, tetrodotoxin was ineffective in blocking the outward current; thereby, showing that baclofen directly affects DMV neurons. This direct inhibition of DMV neurons by baclofen was suggested to reduce inhibitory NANC output to the stomach (Browning and Travagli, 2001), which led us to explore this idea in an *in vivo* rat model. Of particular interest to us was whether the *in vitro* effects of baclofen on DMV neurons would translate to the *in vivo* effects on gastric tone and motility via the DMV-NANC pathway from the nucleus.

Microinjection of baclofen in the DMV increased both tone and motility, which were blocked by atropine methyl bromide. However, the use of atropine methyl bromide as proof of the absence of a NANC pathway is problematic as blockade of muscarinic receptors on gastric smooth muscle can cause further gastric inhibition that can potentially lead to the “floor effect” phenomenon. Indeed, Cruz et al. (2007) administered atropine methyl bromide (0.1 mg/kg, iv) to six rats. Atropine methyl bromide decreased gastric tone (−0.4 + −0.1 mm/Hg, *P* < 0.05) and reduced the amplitude of phasic contractions. In one rat, there was no change in tone after atropine methyl bromide administration. We addressed this potential problem in two of our earlier studies and found that atropine does not limit observations of further gastric inhibition. In these studies (Herman et al., 2009, 2010), we administered atropine methyl bromide systemically to rats and observed the expected decrease in gastric motility. At the nadir of the atropine methyl bromide response, we then intravenously administered nitroprusside and observed an additional significant decrease in gastric tone.

Microinjection of glutamate (500 pmol/30 nl) into the DMV, in the presence of bethanechol infusion (30 μg/kg/min), failed to affect gastric tone and motility (Cruz et al., 2007). This was not the case when nitroprusside was infused to cause gastric relaxation — which it did; thus, confirming that our failure to observe a NANC-mediated transmission was not due to a profound gastric relaxation (Cruz et al., 2007). Additionally, neither atropine nor vagotomy led to the “un-masking” of a gastric relaxation response; indeed, often an increase in intragastric pressure was observed (Cruz et al., 2007).

In the *in vitro* brain slice preparation, baclofen has been reported to act directly on DMV neurons to produce inhibition (Browning and Travagli, 2001). However, if this were the predominant case, and assuming that ongoing activity of these DMV neurons results in the discharge of preganglionic vagal cholinergic nerves innervating gastric smooth muscle, the outcome of a postsynaptic inhibitory action *in vivo* would be a decrease in vagal nerve discharge. Instead, the opposite occurs as baclofen given systemically increases the vagus nerve discharge (Goto et al., 1985; Yamasaki et al., 1991). While Browning and Travagli (2001) accept the findings that baclofen given systemically increases gastric motility and tone; however, they attribute these effects to the postsynaptic inhibition of DMV neurons that provide an inhibitory drive (i.e., ‘NANC drive’) to the stomach. This explanation is difficult to reconcile given that systemic baclofen increases vagus nerve discharge (Goto et al., 1985; Yamasaki et al., 1991). Instead, it is plausible that the potential excitatory effect on DMV neurons due to their presynaptic disinhibition from NTS neurons observed in the *in vitro* rat brain slice preparation (Brooks et al., 1992) is counterbalanced by the direct inhibitory effect of baclofen on DMV neurons (Browning and Travagli, 2001). As suggested by Brooks et al. (1992), the effect of baclofen varies the level and quality of ongoing synaptic input in the dorsal vagal complex (DVC) with its presynaptic action being more relevant than the postsynaptic one to its excitatory role in gastric motility and tone.

**FIGURE 6:**
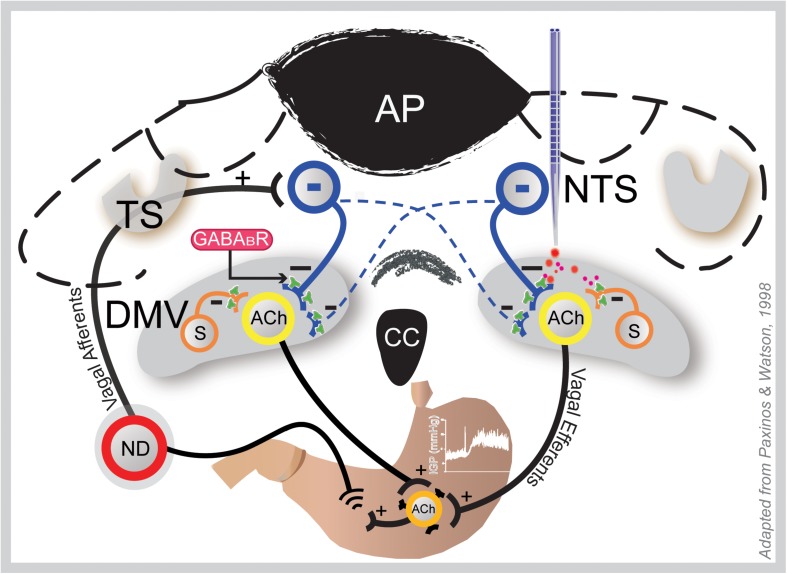
Coronal section of the hindbrain at the level of the area postrema illustrating how GAABA_B_ signaling in the DMV may influence gastric motility and tone. Gastric sensory information from the periphery is conveyed to the NTS via afferent fibers of the vagus that originate in the nodose ganglia (ND; only one shown here). Following integration at the NTS, this information is conveyed back to the stomach via vagal efferent neurons in the DMV; thus, making a reflex loop. Baclofen at the DMV (right side) acts at presynaptic GABA_B_ receptors of the inhibitory NTS neurons (blue) that bilaterally terminate there and local somatostatin GABA interneurons (S), thereby uncoupling cholinergic neurons that project to the gastric musculature from inhibition; hence, causing an increase in gastric tone and motility. This effect is blocked by ipsilateral vagotomy. ACh, acetylcholine; AP, area postrema; DMV, dorsal motor vagal nucleus; NTS, nucleus tractus solitarius; TS, tractus solitarius.

Our data on the excitatory effect of baclofen on gastric tone and motility is in agreement with reports of increases in vagal nerve discharge (Goto et al., 1985; Yamasaki et al., 1991). At the DMV, the drug indirectly excites gastric projecting neurons thereby causing an increased release of acetylcholine at the gastric smooth muscle neuroeffector junction. We suggest that baclofen at the DMV acts at presynaptic GABA_B_ receptors of inhibitory neurons that terminate on gastric projecting neurons in the nucleus (Sivarao et al., 1998; Ferreira et al., 2002, 2005; Pearson et al., 2007, 2011; Lewin et al., 2016). As shown in Figure 6, suppression of this inhibitory output by baclofen would lead to excitation of DMV output neurons (i.e., result in disinhibition). For instance, baclofen acts presynaptically to inhibit GABA (see e.g., Harvey and Stephens, 2004; Chen and Yung, 2005). Indeed, presynaptic GABA_B_ receptors appear to be many folds more sensitive than postsynaptic GABA_B_ receptors (Chen et al., 2017). In the rat ventrolateral periaqueductal gray, 0.01 μM baclofen is required to inhibit presynaptic GABA_B_ receptors (Chen et al., 2017), whereas in the DMV the EC50 reported for the drug to exert its postsynaptic effect is 3 μM (Browning and Travagli, 2001).

The notion that baclofen activates a cholinergic excitatory path is supported by the data of others who tested the effect of GABA_B_ receptor agonists on the efferent neural discharge of the vagus in rats (Goto et al., 1985; Yamasaki et al., 1991). In these studies, GABA_B_ agonist drugs including baclofen produce neural discharges in efferent vagal fibers, which were associated with increases in gastric acid secretion (Goto et al., 1985; Yamasaki et al., 1991; Browning and Travagli, 2001). These data further reiterate (albeit indirectly) the absence of NANC neurotransmission to the stomach. That the DMV-NANC pathway had no role in gastric relaxation was further confirmed by our study in the ferret wherein the role of NANC neurotransmission was evident only for the lower esophageal sphincter (LES), but not for the stomach (Niedringhaus et al., 2008).

Similar to the microinjection in the DMV of the rat, previously it has been reported that intravenous administration of baclofen (0.5 mg/kg) in the anesthetized ferret also evokes an increase in gastric tone (Andrews et al., 1987) as measured via an intragastric balloon. This baclofen-induced increase in gastric corpus tone was apparent in the presence of atropine and elimination of the sympathetic nervous system (i.e., greater splanchnic nerve resection, adrenalectomy, and guanethidine). These results led the authors to propose that gastric pressure was increased by baclofen in part by a reduction in the tonic vagal drive to the intramural NANC inhibitory neurons of the gastric corpus region. While our studies confirmed that baclofen (0.5 mg/kg; IV) increases gastric tone in the ferret; however, our results differ from those previously reported (Andrews et al., 1987) in that atropine methyl bromide completely blocked the response. Based on this inhibition, we conclude that it is highly unlikely that baclofen induces its effect on gastric tone in part by reducing NANC inhibitory activity to the stomach. Nevertheless, it is reasonable to assume the discrepancy between our study and that previously reported (Andrews et al., 1987) may relate to the volume of fluid used in the IGP balloon to generate a baseline gastric pressure. Our volume to distend the stomach was 10 ml; whereas, the volume used in the earlier study was 20 ml (Andrews et al., 1987). It is possible that the engagement of a NANC pathway operating through the DMV depends on the strength of the gastric distention stimulus.

While we have been open to the possibility that a DMV-NANC pathway plays a significant role in controlling gastric motility; our efforts (including the current approach with baclofen) to establish its existence to the stomach has been to no avail (Browning and Travagli, 2001; Ferreira et al., 2002, 2005; Shi et al., 2005; Cruz et al., 2007; Herman et al., 2008, 2009, 2010; Richardson et al., 2013). This is contrary to our findings in the lower esophageal sphincter, where we have provided strong evidence for its existence (Rossiter et al., 1990; Niedringhaus et al., 2008) and at the pyloric sphincter (Richardson, 2012). Hence, based on these observations, we are compelled to conclude that a DMV-NANC pathway has little (if any) physiological relevance for controlling gastric smooth muscle function, apart from sphincter function. Consistent with our findings are the findings of others who have shown that excitation of vagal efferent nerves caused by gastric distension inhibits more than 85% of the DMV afferent neurons (McCann and Rogers, 1992; Zhang et al., 1998; Zhang and Fogel, 2002). If a DMV-NANC pathway was involved, then a significant percentage of DMV neurons should have been inhibited. That they did not, firmly argues against the presence of a gastric NANC pathway originating from the DMV.

In agreement with our findings that the vagus nerve provides only excitatory parasympathetic drive to gastric smooth muscle (non-sphincter) are data that also show IV atropine abolishes gastric motility induced by administration of water into the larynx. This data in conjunction with recordings from vagal DMV neurons (10 caudal and 36 intermediate neurons in the nucleus) showed that their firing rate decreased in response to the administration of water into the larynx (Kobashi et al., 2002). Furthermore, in a recent study, the inhibitory effect of dopamine microinjected into the dorsal vagal complex (DVC) on gastric tone and motility was abolished by ipsilateral vagotomy and attenuated by IV administration of atropine (Anselmi et al., 2017). Intravenous administration of the NO synthase inhibitor, L-Name did not affect the dopamine-induced decrease in gastric tone and motility, which led the authors to conclude that the inhibition was via the disengagement of an excitatory cholinergic pathway (Anselmi et al., 2017).

In summary, our data, as well as those of other investigators demonstrate the functional relevance of a DMV-cholinergic-cholinergic excitatory pathway controlling gastric motility and tone, with little, if any physiological role for the DMV-NANC pathway.

## Data Availability

The datasets generated for this study are available on request to the corresponding author.

## Ethics Statement

All procedures were conducted in conformity with the National Institute of Health (USA) guidelines for the ethical use of animals in research and with the approval of the Animal Care and Use Committee of Georgetown University.

## Author Contributions

MC and EM performed the experiments. MC, NS, and RG conceived and designed the study. MC, GD, and NS analyzed the data and prepared the figures. MC, GD, SV, NS, and RG wrote the manuscript. All authors approved the final version of the manuscript.

## Conflict of Interest Statement

The authors declare that the research was conducted in the absence of any commercial or financial relationships that could be construed as a potential conflict of interest.
